# Solvothermal synthesis of SnO_2_ nanoparticles for perovskite solar cells application

**DOI:** 10.3389/fchem.2024.1361275

**Published:** 2024-01-29

**Authors:** Haixia Xie, Wenxiu Que

**Affiliations:** ^1^ School of Science, Xi’an University of Architecture and Technology, Xi’an, China; ^2^ Electronic Materials Research Laboratory, Key Laboratory of the Ministry of Education, School of Electronic Science and Engineering, Institute of Advanced Energy Storage Electronic Materials and Devices, Xi’an Jiaotong University, Xi’an, China

**Keywords:** solvothermal synthesis, tin oxide nanoparticle, electron transport layer, perovskite solar cell, low temperature

## Abstract

Perovskite solar cells show great potential application prospects in the field of solar cells due to their promising properties. However, most perovskite solar cells that exhibit excellent photovoltaic performance typically require a carrier transport layer that necessitates a high-temperature annealing process. This greatly restricts the scalability and compatibility of perovskite solar cells in flexible electronics. In this paper, SnO_2_ nanoparticles with high crystallinity, good dispersibility and uniform particle size distribution are first prepared using a solvothermal method and dispersed in n-butanol solution. SnO_2_ electron transport layers are then prepared by a low-temperature spin coating method, and the photovoltaic characteristics of perovskite solar cells prepared with different SnO_2_ nanoparticles/n-butanol concentrations are studied. Results indicate that the rigid perovskite solar cell achieves the highest power conversion efficiency of 15.61% when the concentration of SnO_2_ nanoparticles/n-butanol is 15 mg mL^−1^. Finally, our strategy is successfully applying on flexible perovskite solar cells with a highest PCE of 14.75%. Our paper offers a new possibility for large-scale preparation and application of perovskite solar cells in flexible electronics in the future.

## 1 Introduction

Since the initial report in 2009, perovskite solar cells have shown a wide range of applications in the optoelectronics field such as solar cells, light-emitting diodes, phototransistors, and photodetectors in just over a decade due to their high power conversion efficiency (PCE), ease of solution preparation and low cost (Kojima et al., 2009; Xie et al., 2020; Cao et al., 2022; Zhang et al., 2023a; Zhang et al., 2023b; Zhuang et al., 2023). The PCE of perovskite solar cells has increased significantly from the initial 3.8% to the current 26.1%, suggesting promising practical applications for perovskite solar cells in the near future (Kojima et al., 2009; Kim et al., 2012; Xiao et al., 2017; Cui et al., 2019; Cao et al., 2023; Laboratory, 2023; Park et al., 2023).

Currently, most of the initial studies on high-efficiency perovskite solar cells include a carrier transport layer that requires a high-temperature annealing process (>450°C). This process can effectively decrease the lattice defects of the carrier transport layer, enhance the carrier transport efficiency, and reduce the recombination rate. For example, in 2021, Changduk Yang et al. used two fluorinated isomeric analogues and commonly used TiO_2_ with a high temperature annealing process as the hole and electron transport layers, respectively. As a result, a FAPbI_3_ perovskite solar cell has achieved a certified PCE of up to 24.8% (Jeong et al., 2020). In 2023, Peng Gao et al. prepared a TiO_2_ electron transport layer, followed by an annealing process at a high temperature of 450°C for 1 h to fully condense the inorganic network, crystallize the anatase phase, and remove all surfactant residues. As a result, the fabricated Cs_0.05_ (MA_0.17_FA_0.83_)_0.95_Pb(I_0.83_Br_0.17_)_3_ based hole transport layer-free perovskite solar cells achieved a PCE of 11.08% (Lalpour et al., 2023). High temperature processes increase the production cost and complexity of perovskite solar cells, which hinders their industrial application. Additionally, most flexible devices are fabricated on polymer substrates, such as poly (ethylene 2,6-naphthalate (PEN) or polyethylene terephthalate (PET), which cannot withstand high temperatures. This seriously impedes the application of perovskite solar cells in the flexible electronic field.

Recently, tin oxide (SnO_2_) has become one of the most promising materials for electron transport layers due to its excellent properties. These include wide band gaps (∼3.6–4.5 eV), suitable band gap positions, high electron mobilities (240 cm^2^ V^−1^ s^−1^, which is about 100 times higher than that of TiO_2_), and high carrier concentrations (>10^15^ cm^−3^)(Bai et al., 2023; Xu et al., 2022; zhou et al., 2023). Furthermore, SnO_2_ materials are cost-effective, eco-friendly, and can be produced using various low-temperature solution preparation techniques, including the sol-gel, chemical bath deposition, spin coating, and spraying methods. These methods are highly suitable for creating efficient and expansive flexible devices (Yoo et al., 2021; Bai et al., 2023). By combining a hydrothermal method and a low-temperature spin coating method, Xu et al. prepared high-quality SnO_2_ electron transport layers and a flexible device with a structure of PEN/ITO/SnO_2_/Cs_0.05_FA_0.81_MA_0.14_PbI_2.55_Br_0.45_/Spiro-OMeTAD/Au, achieving an efficiency of up to 18.1% and a certified efficiency of up to 17.27% (Liu et al., 2019). In 2023, Yixin Zhao et al. prepared a SnO_2_ electron transport layer with adjustable performance using a low-temperature chemical bath deposition method. They successfully fabricated SnO_2_ nanocrystals with high crystallinity by using Cl as dopants. These nanocrystals were then dispersed in water to create a dispersion for the fabrication of the SnO_2_ electron transport layer using a low-temperature spin coating method. The electron transport layer of the perovskite solar cells was improved by the addition of Cl-doped SnO_2_ nanocrystals. The highest PCE was −25% (effective area of 0.085 cm^2^) and 20% (mini-module with effective area of 12.125 cm^2^) (Wang et al., 2023). These findings suggest that SnO_2_ has significant potential as electron transport layers in high-performance perovskite solar cells.

In this paper, we report a solvothermal method for fabricating highly crystalline SnO_2_ nanoparticles at low temperatures, which are succussed in use of flexible perovskite solar cells. The solvothermal method produces SnO_2_ nanoparticles with uniform particle size and high crystallinity. These nanoparticles are then dispersed in n-butanol solution to fabricate SnO_2_ electron transport layers and devices. The *J*-*V* curves demonstrate that the rigid perovskite solar device reaches a maximum PCE of 15.61% when the SnO_2_ dispersion concentration is 15 mg mL^−1^. Finally, flexible perovskite solar cells by using 15 mg mL^−1^ of SnO_2_ dispersion is fabricate with a PCE of 14.75%.

## 2 Materials and methods

### 2.1 Materials and chemicals

Glass/ITO and PEN/ITO substrates with sheet resistance of ∼15 Ω □^−1^ were purchased from Yingkou OPV Tech New Energy CO., Ltd., Tin Tetrachloride/dichloromethane (SnCl_4_/CH_2_Cl_2_) solution, Dimethyl sulphoxide (DMSO, C_2_H_6_OS, 99.70%), N, N-dimethylformamide (DMF, C_3_H_7_NO, 99.80%), chlorobenzene (C_81_H_68_N_4_O_8_, 99.80%) and tBP (C_9_H_13_N, 95.00%) were attained from J&K Scientific Ltd (Beijing, China), tert-butanol, n-butanol, acetone, anhydrous ethanol, Li-TFSI (C_2_F_6_LiNO_4_S_2_, 99.95%), acetonitrile (C_2_H_3_N, 99.80%) and silver wires (Ag, 99.90%) were obtained from Sinopharm Chemical Reagent Co., Ltd., ammonium methyl iodide (MAI, ≥99.50%), lead iodide (PbI_2_, >99.99%) and Spiro-OMeTAD (C_81_H_68_N_4_O_8_, 99.80%) were supplied by Xi’an p-OLED. All of them were used as received otherwise special specification.

### 2.2 Solvothermal synthesis of SnO_2_ nanoparticles

In a Teflon-lined autoclave, 10.2 mL of SnCl_4_/CH_2_Cl_2_ and 19.8 mL of 1 M tert-butanol solution were mixed and stirred continuously with a magnetic stirrer for 30 min. The solution mixture was subsequently placed in an oven at 100°C for 24 h. Following the reaction, the solution was transferred to a centrifuge tube and a suitable amount of acetone was added. The mixture was then ultrasonically dispersed for 5 min. Later, the solution was centrifuged and cleaned using a centrifuge for 5 min at a speed of 3,000 rpm. The upper solution was then poured out. After repeating these steps twice, a white precipitate of SnO_2_ nanoparticles was collected.

### 2.3 Preparation of perovskite solar cells

The Glass/ITO and PEN/ITO substrates were first cleaned with deionized water and then ultrasonicated with ethanol, acetone, and ethanol for 15 min. They were then dried with high purity N_2_ gas and treated in a UV-Ozone cleaner for 15 min for further use. SnO_2_ dispersions were prepared using n-butanol as a dispersant with varying mass concentrations of 5, 15, 30, 45, and 60 mg mL^−1^. Following a 30-min ultrasonic dispersion treatment, transparent and colorless dispersions were obtained. The SnO_2_ electron transport layers were then prepared by spin-coating the aforementioned SnO_2_ dispersions on the cleaned substrates. In detail, appropriate amounts of SnO_2_ dispersion were used to prepare samples on substrates. Specifically, SnO_2_ 5, SnO_2_ 15, SnO_2_ 30, SnO_2_ 45, and SnO_2_ 60 were used to describe the corresponding samples prepared by the corresponding solutions. The spin coating process was carried out for 30 s at 3,000 rpm, followed by annealing at 150°C for 30 min.

After cooling down, all the samples were transferred into a glovebox to deposit perovskite and hole transport layers sequentially. 2.8 g of PbI_2_ and 1.0 g of MAI were added to 1.5 mL of DMSO and 3.5 mL of DMF at first. The mixture was then magnetically stirred at 70°C for 12^−^h, and filtered with a 0.45-μm polytetrafluoroethylene (TPFE) filter to obtain the 1.2 M of MAPbI_3_ precursor solution. Perovskite films were prepared using a one-step method, involving spinning the films at 1,000 rpm for 5 s and then at 3,000 rpm for 30 s. Additionally, 130^−^uL of chlorobenzene was added dropwise 13 s before the end of the process. Then the samples were annealed at 70°C for 2^−^min and 100°C for 10 min. Furthermore, the Spiro-OMeTAD hole transport layers were spin-coated onto the perovskite layers at 1,500 rpm for 45 s from a Spiro-OMeTAD precursor solution. To prepare the precursor solution, 17.5 μL of Li-TFSI/Acetonitrile (520 mg mL^−1^) and 28.8 μL of tBP were added to 1 mL of Spiro-OMeTAD/Chlorobenzene solution (72.3 mg mL^−1^). The mixture was stirred in a glovebox for 12 h and filtered through a 0.45-um TPFE filter before use.

Finally, the samples were carried out from the glovebox to evaporate a silver counter electrode on top of the Spiro-OMeTAD layers. The active area of each device was defined to 0.07 cm^2^ using customized masks.

### 2.4 Characterization and measurements

The morphology and crystallinity of the samples were characterized by using a transmission electron microscope (TEM, JEM-2010, JEOL Inc., Japan) and a D/max-2400 X-ray diffraction spectrometer (XRD, Rigaku, Japan) with Cu Ka radiation and operated at 40 kV and 100 mA. The surface morphologies of the samples were charactered by a scanning electron microscopy (SEM, JSM-6390, JEOL Inc., Japan) and atomic force microscopy (AFM, SPM-9700HT AFM, Shimadzu, Japan).

All the following measurements were conducted in the ambient environment. The *J*-*V* curves of the devices were measured by using a PVIV-201V I-V Station (Newport Oriel), with a scanning rate of 100 mV s^−1^. The illumination source was calibrated by a Newport 91150V reference cell. The external quantum efficiency (EQE) spectra were tested using a Qtest Station 1000ADX system (Growntech. Inc.) without bias light.

## 3 Results and discussion

**FIGURE 1:**
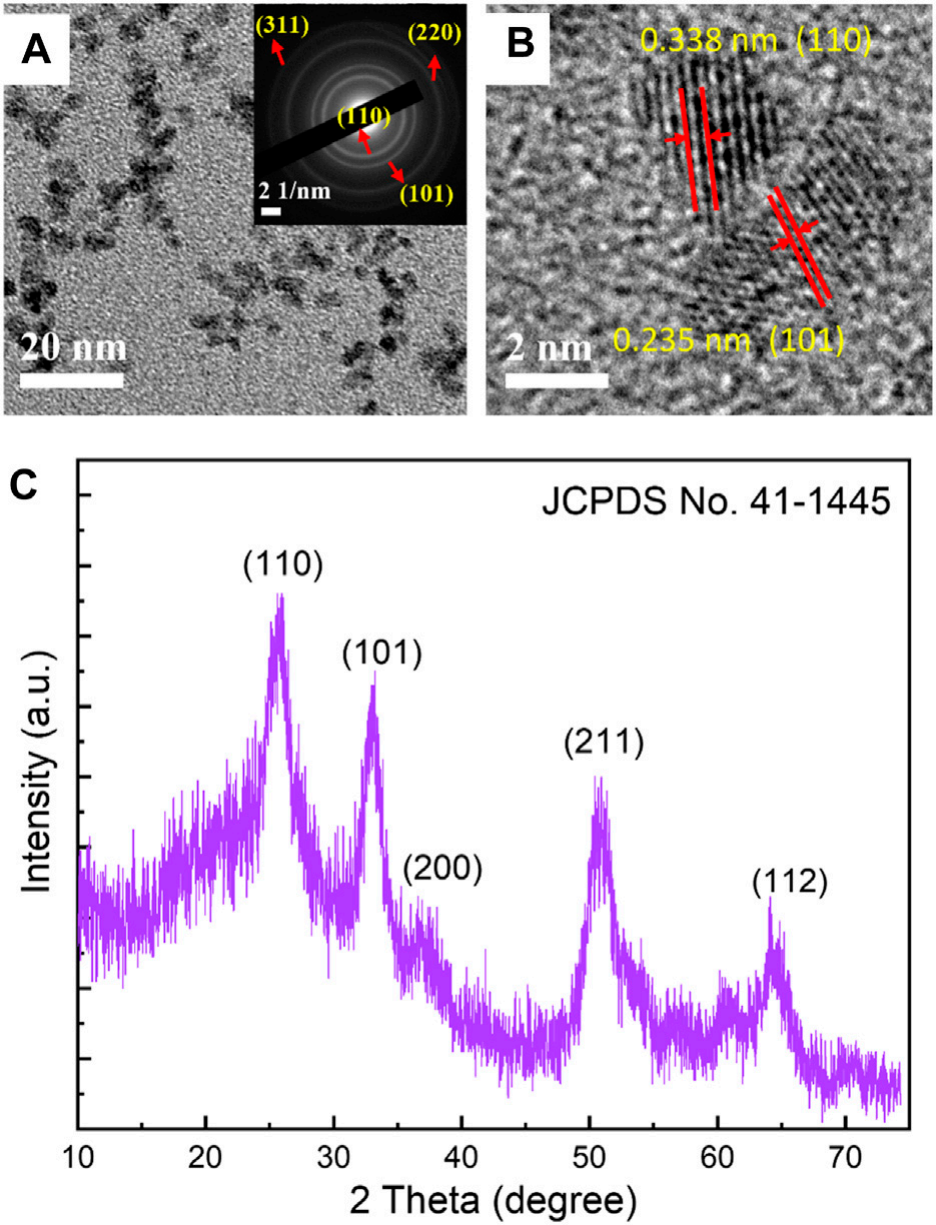
**(A)** Low magnification TEM image (The inset is the corresponding SAED pattern of the SnO_2_ nanoparticles), **(B)** HRTEM image and **(C)** XRD diffraction pattern of SnO_2_ nanoparticles prepared by solvothermal method.

Figure 1A displays the low-resolution TEM findings of SnO_2_ particles. The results show the SnO_2_ have an average size distribution of 4–6 nm. The selective area electron diffraction (SAED) of Figure 1A at the upper right corner indicates that the SnO_2_ are well-crystallized, with four clear diffraction circles corresponding to the (110), (101), (220), and (311) planes of SnO_2_ crystal (JCPDS Card: 41-1445) can be seen.

From the high-resolution TEM (HRTEM) image in Figure 1B, it is possible to distinguish two grains with different interplanar distances. Clear lattice fringes with an interplane d-spacing of 0.343 and 0.235 nm corresponding to the (110) and (101) planes of the SnO_2_ crystal (JCPDS Card: 41-1445) can also be seen, indicating a highly crystalline property for the SnO_2_ nanoparticles. Figure 1C shows the XRD diffraction pattern of the SnO_2_ nanoparticles. Five distinct diffraction peaks appear at 2θ = 26.6^o^, 34.0^o^, 37.9^o^, 51.6^o^, and 64.7^o^, which can be assigned to the (110), (101), (200), (211), and (112) planes of SnO_2_, respectively. No noise peaks are found, indicating the good crystallinity of the SnO_2_ nanoparticles. The high crystallinity of SnO_2_ nanoparticles indicates that there are few defects in SnO_2_, which will be beneficial in achieving high carrier mobility when used as electron transport layers. The crystal grain size is calculated to be −5 nm using the Debye-Scherrer formula, which is in good agreement with the TEM particle size shown in Figure 1A (Li et al., 2017). These well-fabricated SnO_2_ crystalline nanoparticles can fill the valleys among the ITO grains, providing a smooth surface. Additionally, using these highly crystallized SnO_2_ nanoparticles can result in electron transport layers with a high carrier extraction rate and without the need for a high-temperature annealing process.

**FIGURE 2:**
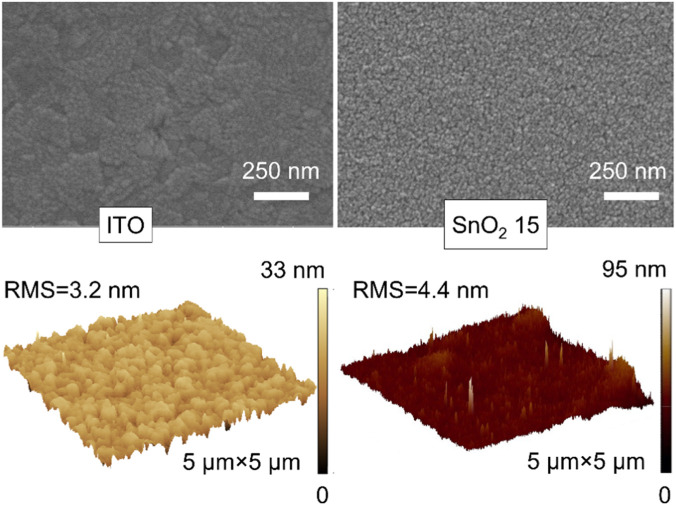
Top view SEM (top row) and 3D-AFM (bottom row) images of the bare ITO substrate and ITO substrate coated with SnO_2_ 15 layer.

Figure 2 compares the surface properties of the ITO substrate and the SnO_2_ electron transport layer (abbreviated as SnO_2_ 15) prepared with a 15 mg mL^−1^ SnO_2_/n-butanol solution as a precursor on a bare ITO substrate, using SEM and AFM images. It can be seen that the surfaces of ITO and SnO_2_ 15 are both smooth and flat, and the surface of SnO_2_ 15 shows a polished and compacted layer of particulate material, which is different from the flake morphology of ITO caused by the deposition of SnO_2_ nanoparticles on the surface of ITO. The smooth, flat and dense SnO_2_ electron transport layer is conducive to the deposition and carrier transport of perovskite films.

**FIGURE 3:**
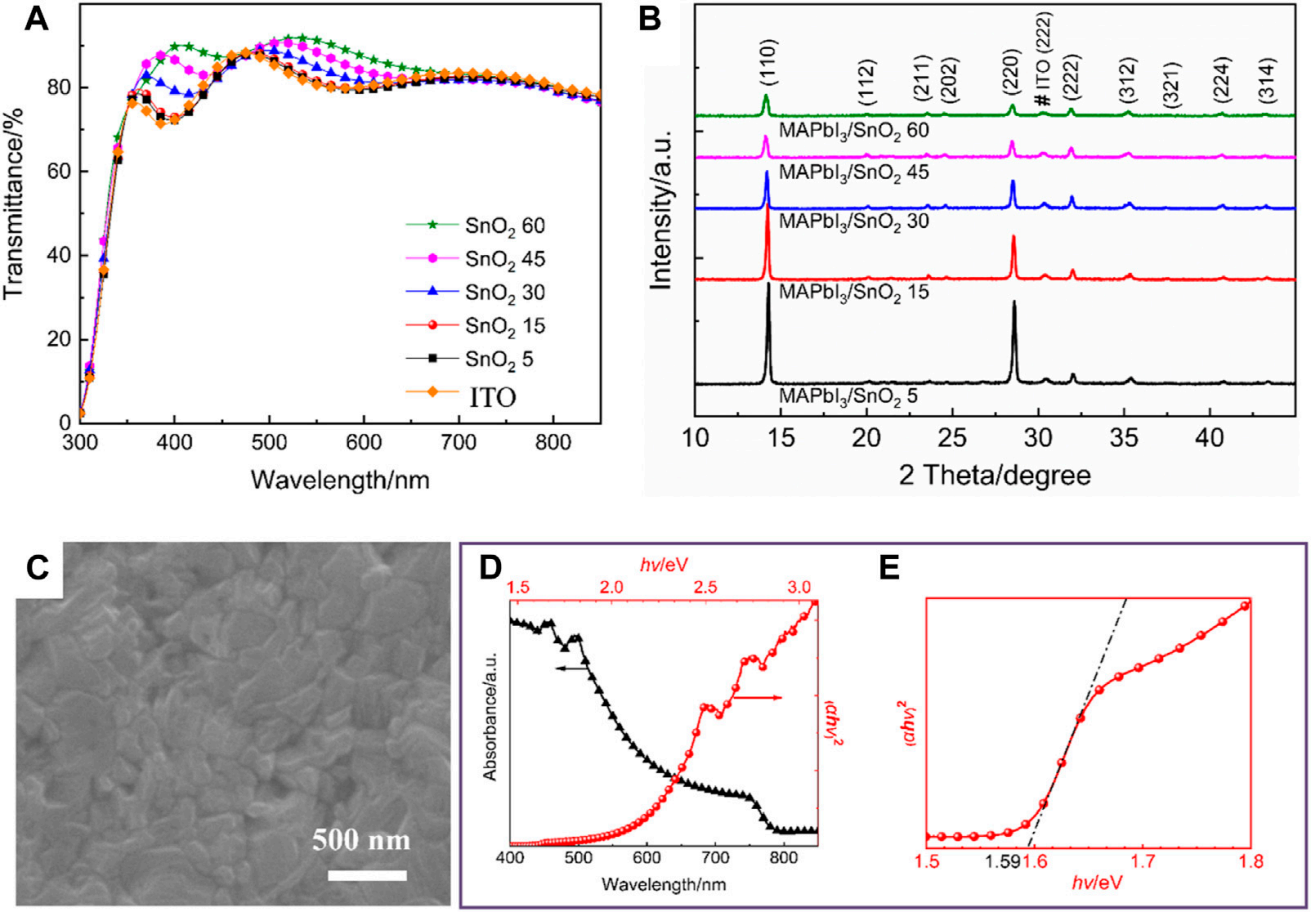
**(A)** Transmission spectra of different samples, **(B)** XRD patterns of MAPbI_3_ perovskite films deposited on different samples, **(C)** SEM images, **(D)** absorption spectra and Tauc plot of MAPbI_3_ perovskite films deposited on SnO_2_ 15 sample, **(E)** partial enlargement of Tauc plot.

In order to understand the influence of the SnO_2_ electron transport layers obtained by different dispersion concentrations on the light transmittance, UV-vis transmission spectrum analysis was performed on SnO_2_ 5, SnO_2_ 15, SnO_2_ 30, SnO_2_ 45, and SnO_2_ 60 samples, as well as on the ITO substrate. As shown in Figure 3A, all of the samples show good transparency in the main absorption wavelength range of perovskite film (380–800 nm), which is beneficial to the light absorption of perovskite films.

The crystallinity, surface morphology, absorption and band gap of perovskite films have an important influence on the photovoltaic performance of planar heterojunction perovskite solar cells. Therefore, the effect of SnO_2_ precursor concentration on the crystallinity of perovskite films was investigated. Perovskite films were spin-coated on the surface of the electron transport layers prepared by SnO_2_ precursor solutions with different concentrations, which were then characterized by XRD, and the results are shown in Figure 3B. All the perovskite films show no impurities and are well crystallized, and the positions of the diffraction peaks of all the films are basically the same, with the three strongest diffraction peaks located at 2θ = 14.1°, 28.5°, and 31.9°, respectively, corresponding to the (110), (220), and (222) planes of the tetragonal perovskite structure, respectively (Guo et al., 2016). As the concentration of the SnO_2_ precursor increases, the diffraction peak intensity of the perovskite films gradually decreases, indicating that the electron transport layers prepared at high concentration are not conducive to the crystallization of the perovskite films.

The surface morphology and UV-vis absorption spectra of perovskite films prepared on SnO_2_ 15 sample are further characterized. Figure 3C shows the top view SEM image of perovskite. It can be seen that the perovskite film is uniform and smooth without any pinholes, which could reduce the recombination of photogenerated carriers and improve the photovoltaic performance of the corresponding devices. Figure 3D shows the UV-Vis absorption spectrum of the perovskite film. It can be seen that the MAPbI_3_ perovskite film has a strong absorption in the short wavelength region. The band gap (*E*_g_) of a semiconductor can be obtained from the Tauc curve (Huxter et al., 2008). As shown in Figure 3E, the band gap of the as-prepared MAPbI_3_ is about 1.59 eV, which is in agreement with the value reported in the literature (Noh et al., 2013; Eperon et al., 2014).

**FIGURE 4:**
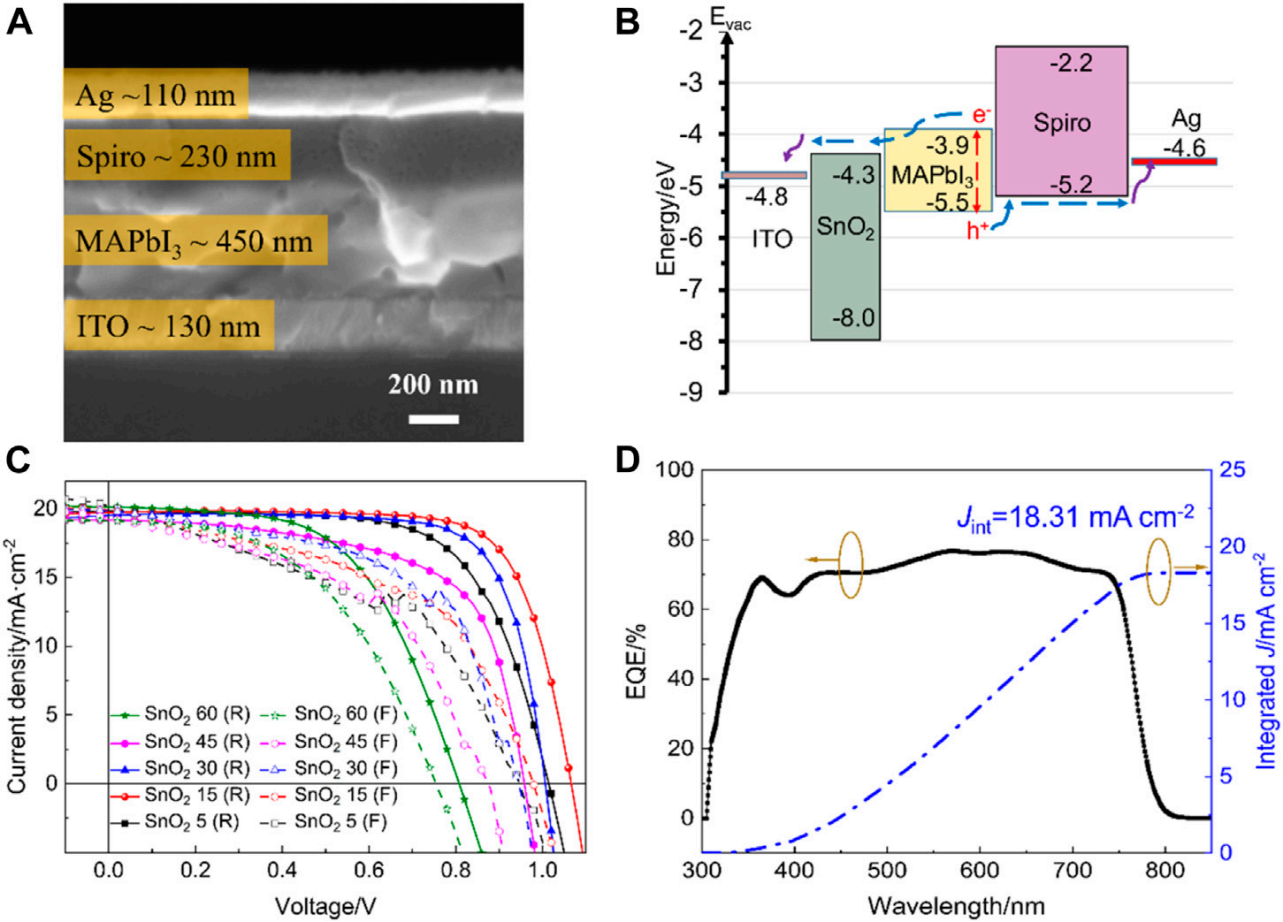
**(A)** Cross-sectional SEM image and **(B)** energy band structure of the perovskite solar cell. **(C)** *J*-*V* curves of the rigid perovskite solar cells based on different SnO_2_ electron transport layers with the highest efficiencies. **(D)** EQE spectra and integrated *J*_sc_ from EQE spectra of the rigid perovskite solar cell based on SnO_2_ 15 electron transport layer.

The cross-sectional SEM image of the SnO_2_ 15 electron transport layer-based device is used as a representative sample to characterize the device, and the result is shown in Figure 4A. The structure of the device can be listed as Glass/ITO/SnO_2_/MAPbI_3_/Spiro-OMeTAD/Ag, showing that the different layers of the device are tightly bonded without any no cracks. The thickness of the ITO, MAPbI_3_, Spiro-OMeTAD and Ag is approximately 130, 450, 230, and 100 nm, respectively. It is important to emphasize that the SnO_2_ electron transport layer is too thin to be observed. The schematic energy level diagram for the SnO_2_ electron transport layer device is shown in Figure 4B, which shows a well-established energy level alignment (Jiang et al., 2010; Noh et al., 2013; Jaramillo-Quintero et al., 2015; Zhu et al., 2017).

In order to optimize the concentration of the SnO_2_ nanoparticle dispersion, SnO_2_ electron transport layers and corresponding rigid devices fabricated with different concentrations of SnO_2_ nanoparticle dispersion are prepared, with the *J*-*V* curves of the highest efficiency shown in Figure 4C. “R” stands for reverse scanning and “F” stands for forward scanning. The parameter values for the relevant devices are summarized in Table 1. It can be seen that when the concentration of the dispersion was 5 mg mL^−1^, the corresponding devices obtained efficiencies of 13.38% and 9.28% under reverse and forward scanning, respectively, and when the concentration was increased to 15 mg mL^−1^, the two efficiencies were increased to 15.61% and 10.70%, respectively. However, as the concentration increases, the efficiency of the corresponding device in both forward and reverse scanning decreases, in particular, the open-circuit voltage (*V*_OC_) and fill factor (FF) decrease significantly. This may be due to the fact that, on the one hand, the films obtained with a high concentration of SnO_2_ are thicker, which is not favorable for fast charge carrier transport and, on the other hand, the crystallinity of the perovskite films on the thicker SnO_2_ films is also poorer and recombination is more severe. Therefore, 15 mg mL^−1^ was found to be the optimum concentration of SnO_2_ dispersion.

**TABLE 1:** Photovoltaic parameters for the rigid devices according to Figure 4C.

ETLs	Scanning direction	*V*_OC_/V	*J*_sc_/mA cm^-2^	FF	PCE/%
SnO_2_ 5	R	1.01	19.79	0.67	13.38
	F	0.95	20.09	0.49	9.28
SnO_2_ 15	R	1.06	19.72	0.74	15.61
	F	0.99	19.81	0.54	10.70
SnO_2_ 30	R	1.01	19.50	0.75	14.67
	F	0.94	19.76	0.58	10.85
SnO_2_ 45	R	0.96	19.17	0.63	11.53
	F	0.87	19.27	0.51	8.44
SnO_2_ 60	R	0.81	20.12	0.56	9.14
	F	0.75	19.09	0.49	7.07

The above results also show that the planar heterojunction devices based on SnO_2_ obtained by the solvothermal method have a significant hysteresis, which is mainly due to the large number of defects in the interior of the perovskite film, in the region close to the surface and at the interface, These act as traps for the electrons and holes and are rapidly filled under reverse scanning conditions to form a good contact at the interface, whereas under forward scanning the efficiency obtained from the test at this point is low due to the fact that the transport of charge is partially trapped by the defects and hence hysteresis occurs.

Figure 4D shows the EQE spectra for the rigid device based on the SnO_2_ 15 electron transport layer. It can be seen that the initial threshold wavelength of the EQE spectrum for the device is at −780 nm, which is consistent with the band gap of 1.59 eV for the perovskite film. The device has a high photoelectric response in the wavelength range between 380 and 760 nm, with a maximum EQE of −77% and an integrated current density of 18.31 mA cm^−2^. There is some discrepancy between the integrated current density and the short-circuit current density obtained from the *J*-*V* curve, mainly due to insufficient filling of defects in the perovskite and the electron transport layer in the bulk and at the interface. As the EQE curve is tested under weak monochromatic light irradiation without optical bias, the photogenerated carrier concentration of the perovskite film is much lower than that tested under simulated sunlight irradiation. The lower carrier concentration makes the defects in the bulk and at the interface unable to be fully filled, and the photogenerated carriers produced by weak light are easily recombined at the interface or in the bulk, which reduces the efficiency of interfacial charge collection, resulting in the integrated current density being lower than the short-circuit current density.

**FIGURE 5:**
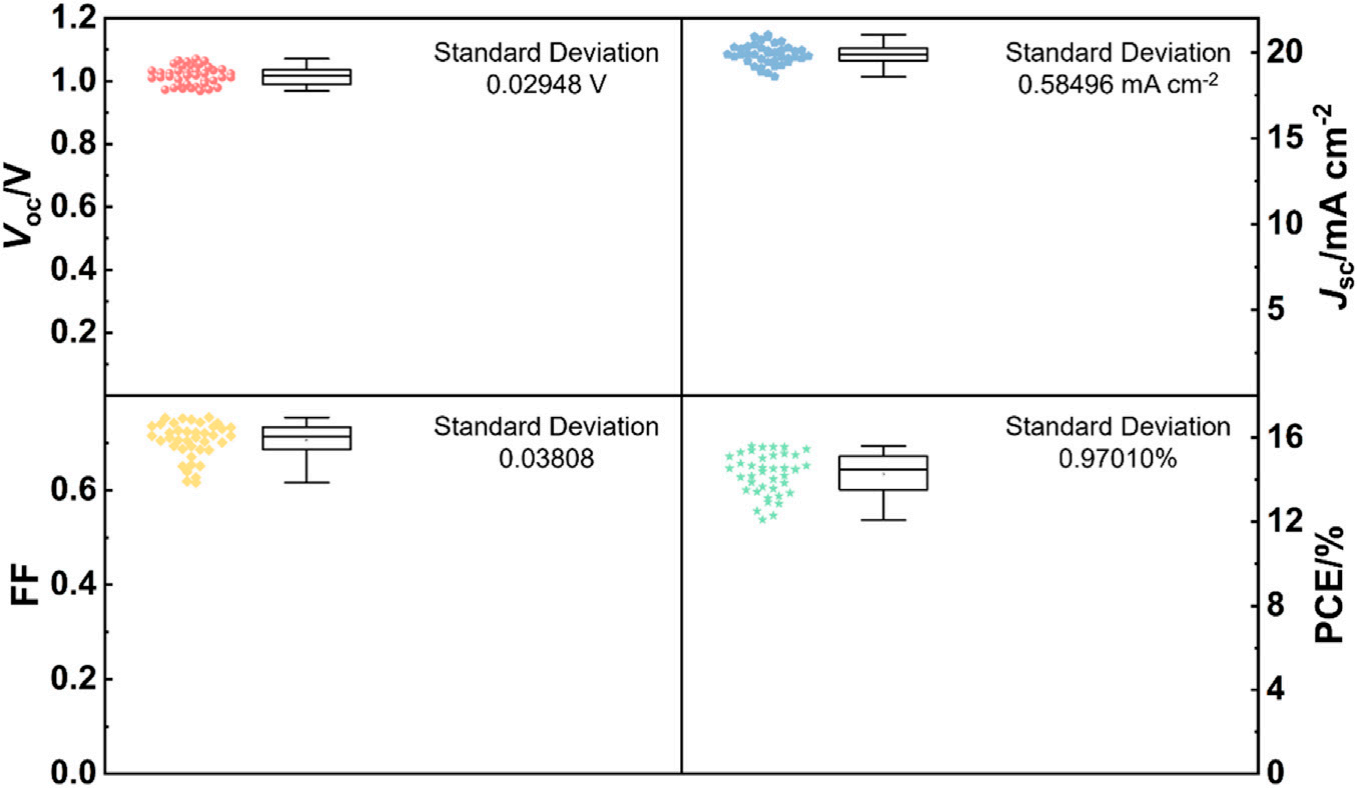
Box chart of photovoltaic parameters (*V*_oc_, *J*_sc_, FF and PCE) obtained from 40 devices of the rigid perovskite solar cells based on SnO_2_ 15 electron transport layer under reverse scan with an AM 1.5G illumination.

**FIGURE 6:**
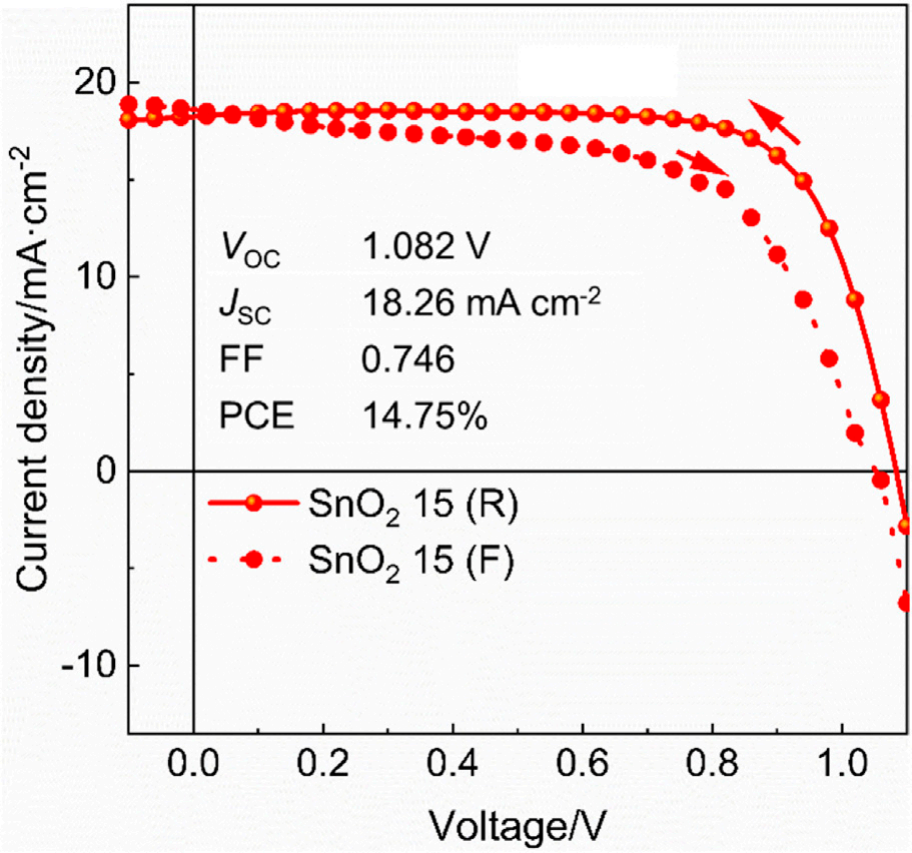
*J*-*V* curves of the flexible perovskite solar cell based on SnO_2_ 15 mg mL^−1^ electron transport layer.

Furthermore, 40 devices of rigid perovskite solar cells based on the SnO_2_ 15 electron transport layer are fabricated and the statistical results of photovoltaic parameters are shown in Figure 5. Clearly, the device performance parameters distribute in a relative narrow range, showing a good reproducibility of our devices. Finally, flexible PEN/ITO was adopted to substitute rigid glass/ITO substrate to fabricate flexible perovskite solar cells at low temperature by using optimized SnO_2_ dispersion (15 mg mL^−1^). The best-performing flexible device has achieved a PCE of 14.75% with a *V*_OC_ of 1.082 V, a *J*_SC_ of 18.26 mA cm^−2^ and an FF of 0.746 under reverse voltage scan (Figure 6). This study is expected to open a new way to prepare SnO_2_ films at a low temperature for high performance flexible devices.

## 4 Conclusion

In this paper, SnO_2_ nanoparticles with high crystallinity, good dispersibility and uniform particle size distribution were prepared by a solvothermal method. SnO_2_ electron transport layers and perovskite solar cells were then prepared using different concentrations of SnO_2_/n-butanol dispersions. Although the electron transport layer prepared with a high dispersion concentration shows a better light transmittance, the perovskite film on it has a worse film crystallinity. And a thicker SnO_2_ film can lead to more carrier recombination and a higher internal resistance, resulting in a lower open-circuit voltage and fill factor. Ultimately, the optimal rigid and flexible device was obtained by using a SnO_2_/n-butanol dispersion of 15 mg mL^−1^ with a PCE of 15.61% and 14.75%, respectively. Our study paves a new way to prepare SnO_2_ electron transport layers at a low temperature, making it suitable for flexible solar cells.

## Data Availability

The original contributions presented in the study are included in the article/Supplementary material, further inquiries can be directed to the corresponding authors.
